# Optimisation of the Heterogeneous Joining Process of AlMg3 and X2CrNiMo17-12-2 Alloy by FSW Method

**DOI:** 10.3390/ma16072750

**Published:** 2023-03-29

**Authors:** Anamaria Feier, Ioan Both, Edward Petzek

**Affiliations:** 1 Department of Materials and Manufacturing Engineering, Mechanical Faculty, Politehnica University Timișoara, Bl. Mihai Viteazu No. 1, 300222 Timisoara, Romania; 2 Department of Steel Structures and Structural Mechanics, 1 Ioan Curea Str., 300224 Timisoara, Romania; 3 Department of Steel Structures and Structural Mechanics, SSF Romania, 1 Ioan Curea Str., 300224 Timisoara, Romania

**Keywords:** dissimilar joint, FSW process, milling machine, solid-state joining

## Abstract

This paper presents experimental investigations on the solid-state joint of 3 mm sheets of AlMg3 alloy with X2CrNiMo17-12-2 stainless steel. The study presents a dissimilar joint that was made in a solid state using a modified milling cutter. The study highlights the possibility of using this type of joint in a naval field. The paper presents all the steps of the joining process, from the technological parameters to the examination and numerical validation of the obtained specimens. A numerical model was defined in Abaqus, considering a Static analysis, and the results demonstrated a good similarity with a small discrepancy observed in the elastic range of the specimen behaviour. In the conclusions, this study will provide some recommendations for the optimisation of this joint and proposals for future studies; the idea for this study started from the dissimilar joints used in the naval field. The article also briefly presents some dissimilar joints made on the same milling machine and in the same laboratory.

## 1. Introduction

In the early 1990s, a new joining technology called friction stir welding (FSW) was invented and patented by TWI Cambridge [1]. The procedure was first applied at an industrial level in Sweden, in the year 1995 [1]. Due to its qualities, this procedure brought interest to some economically powerful countries such as USA and Japan.

In 2015, Lucian A. Blaga and his team had developed a special process on Friction Riveting (FricRiveting) as a new joining technique in GFRP lightweight bridge construction; through this process, it has been possible to make joints from different materials for emergency bridges [1].

A current concern in society is the compromise between the benefits of using lightweight materials and how to integrate these into larger multi-material designs projects. The wider the range of possible joining technologies to perform dissimilar joints, the less compromising or restricted might be the usage of these materials [1]. The more traditional and well-established methods to perform connections between different material classes are known as mechanical fastening, but most recently, the FSW process and FSW-derived processes solved a lot of situations. The recent research on this process demonstrated that it can also be applied in civil engineering, namely bridge construction [2].

Analysing, as an example, the case of emergency bridges [1,2], where joints from different materials were proposed, the joint studied in this work is suitable for certain construction areas.

**Figure 1:**
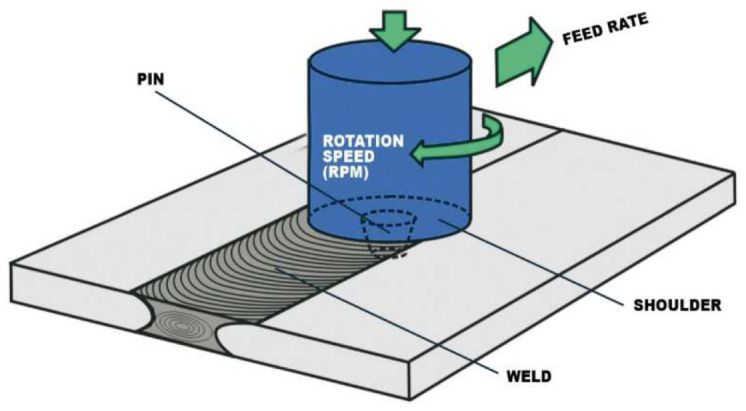
Schematic illustration of friction stir welding [5].

The schematic illustration of friction stir welding is presented in Figure 1 for a butt joint configuration. Friction stir welding (FSW) is a solid-state process, which means that the objects are joined without reaching the melting point. In friction stir welding (FSW), a cylindrical shouldered tool with a profiled pin is rotated and plunged into the joint area between two pieces of sheet or plate material.

Friction stir welding (FSW) relies on heat derived from the friction between the active element and the materials to be welded, heat that produces the softening of the marginal areas of the base materials. Through the advance movement of the tool along the joint, the plasticised material becomes homogenous, and through cooling, a solid-state joint between the base materials is produced [1,2].

The friction stir welding (FSW) process involves a joint formation below the basematerial’s melting temperature. The heat generated in the joint area is typically about 80–90% of the melting temperature [3,4].

There are a variety of friction welding techniques:

Rotary Friction Welding—the most popular type of friction welding and used for parts where at least one piece is rotationally symmetrical such as a tube or bar.

Linear Friction Welding—used for jet engine components, near-net shapes, and more where the limitation on the parts is only based upon the mass of the moving part, not the geometry of the interface.

Friction stir welding—often used for aluminium plates, extrusions, and sheets where seam or butt welds are made between thin components without a restriction on the component length.

There are a variety of types of friction stir welding

- Friction Stir Spot Welding;
- Double-Sided friction stir welding—Stationary Shoulder FSW. [5]

The main features of friction stir welding are:

✓ Friction-based joining procedure.
✓ Solid-state joining process.

Metallurgical advantages of the procedure [5]: There is no melting, reduced deformations, and high dimensional stability and reproducibility. The quantity of alloying elements from the materials does not decrease, there are excellent mechanical properties in the welded joint and fine granulation structure, and there is an absence of the cold cracking phenomenon.

Advantages in terms of environmental protection [5]: There is no need to use protection gas, minimal preparation procedures of the surfaces, and no waste resulted from the operation of polishing; it does not involve the use of solvents and degreasers. There is a low consumption of consumables and absence of harmful emissions. 

Energetical advantages [5]: There is more reduced energy consumption compared to laser welding; there is reduced fuel consumption in automotive, naval, and aeronautical applications because of the low weight of the welded parts; the weight reduction results from the use of improved materials.

Disadvantages of FSW [5]: Tool wear/costs. A great part of the tool wear takes place in the plunging phase; the welding speed for single pass welds is lower for some alloys than for other electric arc welding processes; the equipment used for FSW is massive and expensive due to the high pressing forces; high melting point materials such as steel and stainless steel have some limitations in terms of the welding tool; the absence of an additive material leads to difficulties in producing corner welds; there is a presence of a crater (keyhole) at the end of the welding seam

Friction stir welding can be used in the following industrial domains [5]: Naval and offshore constructions, automotive, railway, aerospace, fabrication, and others (electrical, oil and gas, nuclear industry, and construction). In the area of naval constructions, FSW can be used for [5]: Aluminium panels for freezing fish on fishermen, joining extruded elements for bridges, comb-like panels, and panels that are resistant to salty water.

**Figure 2:**
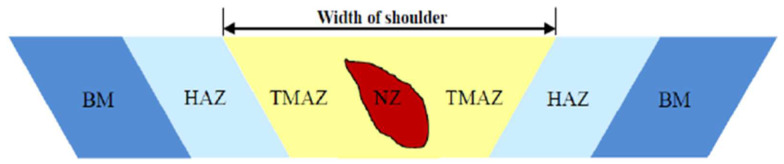
Influence zones of an FSW joint [5].

In Figure 2, the influence zones of a welded joint obtained through the friction stir welding technology are represented [5].

These are as follows:

- BM—base metal;
- HAZ—heat-affected zone;
- TMAZ—thermo-mechanically affected zone;
- NZ—nugget zone.

In the heat-affected zone, the microstructure and mechanical properties are affected by the heat generated during the FSW process, but plastic deformation does not occur.

The thermo-mechanic-affected zone is the one where the material suffers mechanical deformation. It is plastically deformed, and the process is comparable to the hot-metal working [6,7,8,9].

The nugget zone is characterised by intense plastic deformation and heating through friction during the FSW process, which leads to the formation of a fine-grain, recrystallised microstructure. This is the zone formerly occupied by the tool’s pin. The central nugget contains fine grains and is formed by different thickness layers, such as “onion rings” (also known as “metallurgical strips”). This repetitive macroscopically visible model in the cross and side section of the weld is a unique characteristic which appears during FSW and related processes. As a result, the fine-grain microstructure confers excellent mechanical properties, a good fatigue resistance, high deformability, and very good plasticity [7,8].

Specific defects of FSW welding:

(1) Lack of connection
  appears when welding dissimilar materials and if there is insufficient diffusion between the materials.
(2) Lack of penetration
  appears at the inferior base of the sheets, insufficient welding depth, and inadequate welding pin length.
(3) Defects of the closing area—
  uncleanness or traces on the active rotating element negatively influence the aspect of the weld and also the insufficient pressing forceinsufficient pressing force.
(4) Structure defects—
  inadequate thermic regime.

Change of the crystalline grains and the length and pin type.

## 2. Materials and Methods

The method used in this study is intended to highlight the achievement of a dissimilar joint using a basic method and an upgraded milling machine. The joining of the two materials used was carried out in a solid state without one of the materials used reaching the melting point, which is basically using the principles of the FSW process. The milling machine used was upgraded with a pin, which is a tool specific to the FSW process.

### Materials

The materials used in the experiments are:

⮚ AlMg3 (EN AW 5754);
⮚ X2CrNiMo17-12-2.

The AlMg3 alloy is a medium-hardness alloy recommended for welded structures in a nuclear environment, the production of boilers and heaters, applications in a marine environment (boat production), and road signs [10]. 

The X2CrNiMo17-12-2 alloy is characterised by a good intergranular corrosion resistance up to a temperature of 300 °C, good resilience, and heat resistance up to temperatures of 550 °C. It is used in the following industries: pharmaceutical, chemical, food, aerospace, automotive, in construction, and in petrochemistry [11].

The chemical composition and the mechanical characteristics of the AlMg3 alloy are presented in Table 1 and Table 2. Table 3 represents the mechanical properties and the content of alloying elements of the X2CrNiMo17-12-2 stainless steel [11,12].

The infrastructure used for the solid-state joining of the two materials AlMg3 + X2CrNiMo17-12-2 was a milling machine on which a pin made of 56SiCr7 steel was mounted. The pin was crafted on some lab from where the milling machine is [13,14].

Table 3 contains a comparison in terms of several criteria between a milling machine such as the one used in the experiments and some equipment that is usually provided in the processes of friction welding with a rotating active element [15,16].

Table 4 shows the chemical composition of the turned pin’s material.

**Figure 3:**
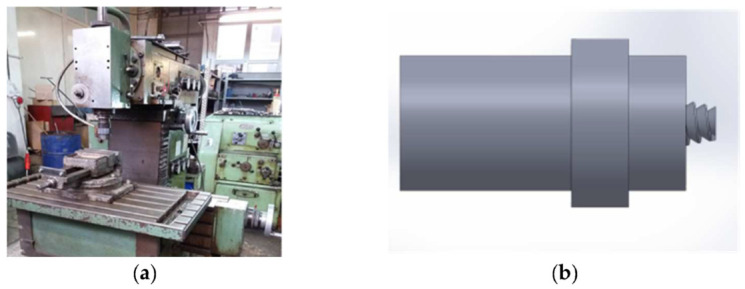
Equipment used for the FSW experiments, consisting of a mill machine with the following characteristics: Speed control (**a**); FSW tool manufactured for the present work—the length of the pin: 2.62 mm (**b**).

**Figure 4:**
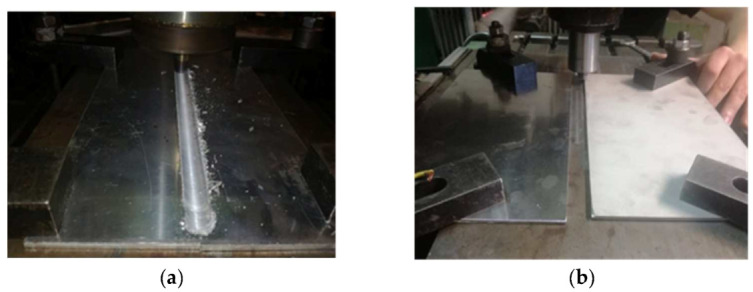
(**a**,**b**) Images with the steps of the joint making.

In Figure 3a,b are shown images illustrating the infrastructure used in the experiments, more specifically, the milling machine and the pin made of 56SiCr7; in Figure 4a,b, the steps of obtaining the joint by the principle of the FSW process are presented [12]. The joint was obtained using a universal milling machine FUS 32. The specifications of the milling machine used for the FSW process are presented in the technology, as shown below. The pin had a cylindrical shape with striations to obtain as much material as possible into the joint.

The joint was obtained by inclining the pin at an angle of 2 degrees to the aluminium so that the softer material can be brought over the harder material, i.e., aluminium over stainless steel. In obtaining this type of joining, it should be observed that the softer material should be brought over the harder one so that the two different materials mix in the contact area.

## 3. Results and Discussion

The joint obtained started from the idea of dissimilar joints used in naval fields (namely TRICLAD joints); the joint was obtained from AlMg3 and X2CrNiMo17-12-2. The procedure of the optimisation of the technology took 3–4 weeks; at the beginning, the technology was optimised on the AlMg3 sheets with the same length of pin and after validation of the technology on AlMg3, the study was carried out on the AlMg3 and X2CrNiMo17-12-2.

The study was carried out starting from the infrastructures available in the laboratory and applying the principles of the FSW process. The aim was to demonstrate that even with a machine from the 1960s, slightly upgraded, it is possible to produce high quality and dissimilar joints. The CNC was equipped with a pin, a tool specific to the FSW process, and with the parameters presented in the next subchapter, the joining was obtained and examined both non-destructively and destructively.

### 3.1. The Process of Joining the AlMg3 + X2CrNiMo17-12-2 Specimens

The research began from the idea of TRICLAD. In the early stages of the research, the same defects appeared in the joint; after the optimisation of the joining technology and the shape of the pin were obtained, the results were presented in this paper.

The dimensions of the parts were 3 × 250 × 350 mm. From the parts (3 × 250 × 350 mm), smaller specimens were sampled for the tensile test and the microscopic tests (can be observed in Section 3.2).

The joining technology optimised used was:

- Milling cutter speed: 1250 rpm;
- Advance speed: 0.40 m/min;
- The angle of inclination of the pin: 2°;
- The length of the pin: 2.62 mm;

The optimisation of the procedure consisted of two stages:

⮚ The first phase was the joining on one side of the AlMg3 + X2CrNiMo17-12-2 alloy.
⮚ The second phase was the joining on both sides of the AlMg3 + X2CrNiMo17-12-2 alloy.

**Figure 5:**
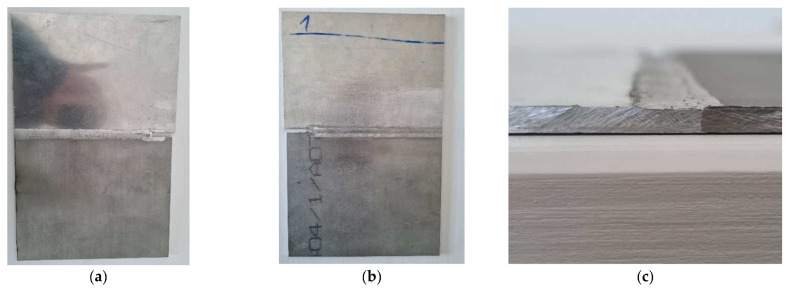
A joining of the AlMg3 + X2CrNiMo17-12-2 alloy; (**a**) first stage was the joining on one side, (**b**) the root of the joining, and (**c**) section of the joint.

It was observed that in the first phase, an area of a lack of penetration resulted. The next step was an optimisation of the welding/joining technology; the team decided to join the parts on both sides, as in Figure 5, which means that after making the joint on one side, the parts were turned on the opposite side using the same welding technology. By joining the parts on both sides, the imperfection (area of lack of penetration) disappeared [12].

As presented above in this article, a joint between the two materials utilised in the naval industry is TRICLADUL, which is an alloy that consists of two layers of aluminium and one of steel. TRICLAD is a special plating, which is generally assigned to the naval domain and facilitates the joining of aluminium structures with steel structures. It is produced in the form of a board with standard dimensions of 1.5 × 4.0 m, the useful surface of which is 1.3 × 3.8 m. Strips or other forms of semi-finished products can be obtained through cutting from it.

**Figure 6:**
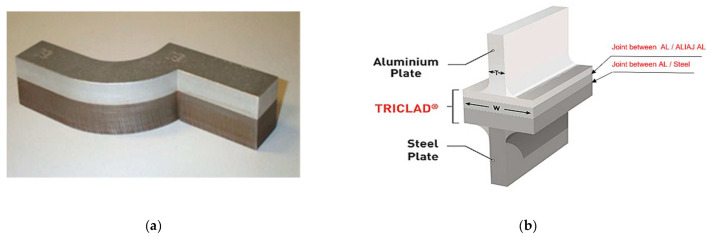
TRICLAD joint used in the naval sector; (**a**) TRICLAD strips; (**b**) TRICLAD composition.

In Figure 6, a part made of TRICLAD and his composition can be observed; this type of material is used often in the naval industry. This material TRICLAD is used as a strip with two sheets positioned vertically [12,17] when a dissimilar joint is needed.

The TRICLAD is used for the idea that the stainless-steel layer will be welded to the underside of the vessel (the area that needs to resist corrosion; a harder, stainless material is required) and the aluminium layer of the TRICLAD composition will be welded to the top of the vessel (the deck—which is made of a lighter material, namely aluminium).

### 3.2. Non-Destructive and Destructive Examination of Specimens

To evaluate the load-bearing capacity of the obtained joints, non-destructive and destructive tests were required to validate the optimised procedure used. Accordingly, to EN ISO 25239-5, the surface defects can be detected by visual inspection (macroscopic examination), apart from insufficient penetration welding and to validate the load-bearing capacity, tensile tests were needed.

The samples were examined:

- Visual;
- Tensile test;
- Microscopy analysis.

The results of the joining were:

- Excessive surface flash formation.
- Flashes are the excessive expulsion of material on the top surface, leaving a corrugated or ribbon-like effect along the retreating side that is generated under a too hot process condition or a too high weld pitch.

Flashes are caused by excessive overpressure or plunge depth; a thickness mismatch between the advancing side and retreating side [18,19] can be observed in Figure 5a.

An excessive lack of fusion may result in a reduction in terms of mechanical properties and can lead to a thinning of the material thickness. Flashes can be observed upon visual inspection. In the visual examination, the specimens fulfil the requirements, the weld was adequate, and the shape of the joint was appropriate [7,8].

**Figure 7:**
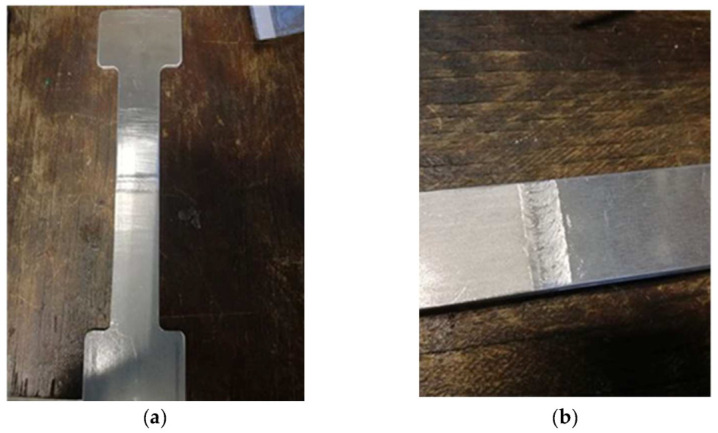
(**a**,**b**) Pictures of specimens prepared for tensile testing.

After visual examination, the next step was tensile tests; in Figure 7a,b are the specimens prepared for the tensile test according to the standards.

**Figure 8:**
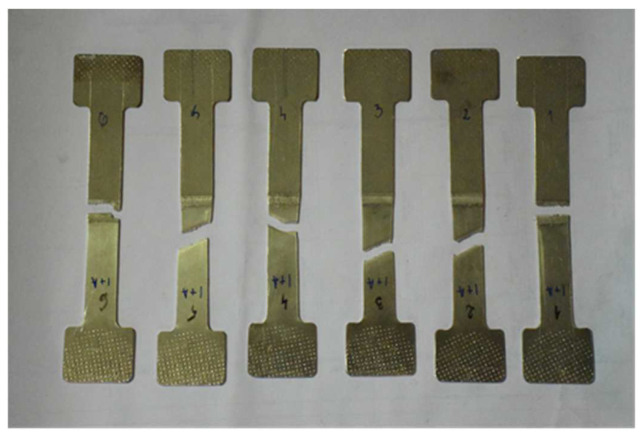
Pictures of the six specimens after the tensile test.

In the second phase, i.e., the destructive testing, the tests demonstrated that two out of six specimens prepared for tensile testing failed in the joint area. The two specimens that failed are the specimens sampled from the end of the piece of the 3 × 250 × 350 mm plate, the area where the weld closure crater is located (a defect specific to this process). Figure 8 provides all six specimens after the tensile tests, and it can be observed that specimen 1 and 6 failed (at the beginning and the end of the piece).

The tensile tests were carried out in the CMMC-UPT department on the TESTWELL/UTS machine. The TESTWELL/UTS tensile testing machine was used, and the machine has the following characteristics:

- Hydraulic pans;
- Tensile tests and compression tests can be performed;
- Maximum force: 250 kN;
- Computer-assisted machine control, acquisition, and post-processing of results.

Table 5 shows the results of the tensile tests performed on the specimens. The lowest yield strength was 227 MPa for specimens 1 and 6. It can be observed that the elongation A_t_ is also significantly lower in the specimens that failed in the welded joint.

**Figure 9:**
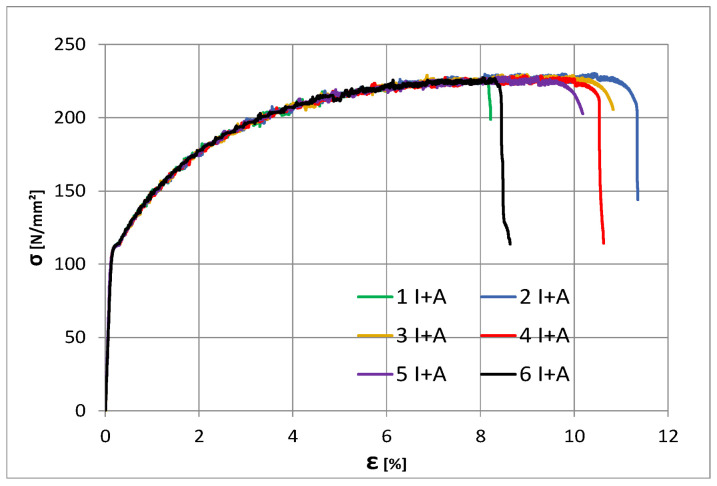
Tensile test curves.

The usual tensile test was performed; specimens were made according to the tensile standard and tensile test parameters were respected, namely speed, time, specimen grips, etc (Figure 9).

As a complement to the tensile tests, some microscopy analyses were also carried out to observe possible areas with a lack of penetration.

**Figure 10:**
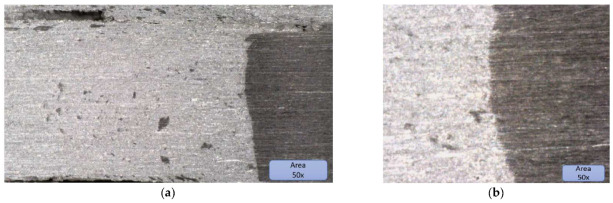

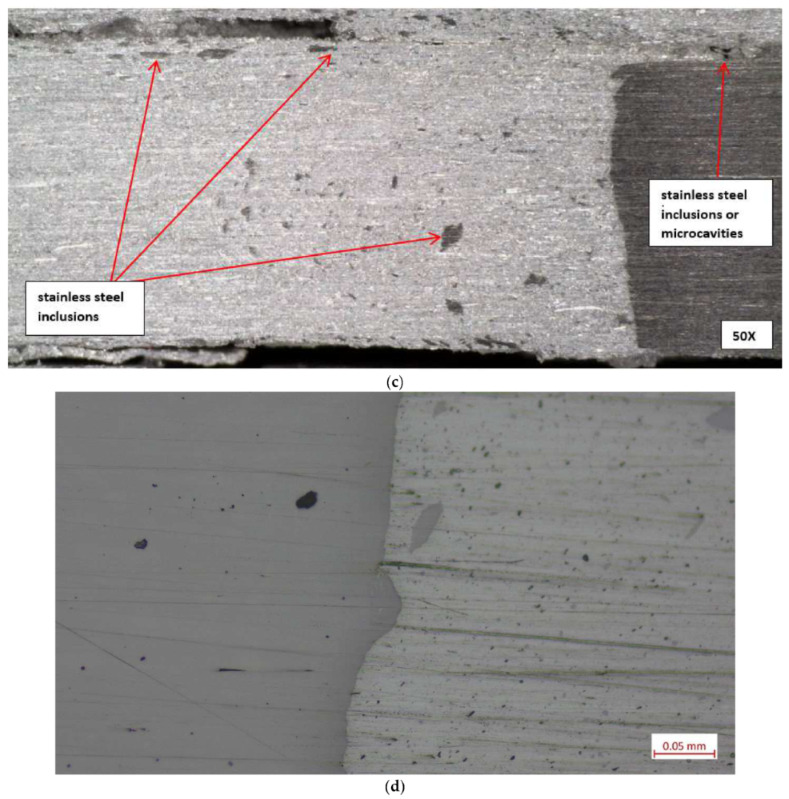
(**a**) Microscopic image of the aluminium area where the stainless-steel inclusions can be observed, (**b**) microscopic images of the joining area, and (**c**) microscopic image with stainless steel inclusions and microcavities. (**d**) Microscopic image at 200× with stainless-steel inclusions.

Microscopic structure of specimens:

Figure 10 shows the microstructure of specimens; the microscopic image presents the joint and interface very well. The two materials can be observed distinctly; the lighter coloured material is AlMg3, and the darker coloured material with a higher hardness is X2CrNiMo17-12-2.

The microscopic structure of the specimens can be observed in Figure 10. In Figure 10a, the inclusions of stainless steel in AlMg3 can be observed. All the dark inclusions in the left surface look less like material adhesions and more like material pull-outs due to the rotating tool. This is an indication of a far from optimal joining technology for the material on the left, which may be a type of cold rolled, heavily roughened rolling structure.

The joint was validated because the failure of joint happened due to the final crater of the joint generated by the riveting of the pin at the beginning and end of the weld (hypothesis validated by tensile tests). The line of joint between the two materials is very well highlighted, and the meshing between them and the sealing of the joint with the softer material, namely AlMg3, can be observed

From a process point of view, the two materials do not reach the melting point; however, only in the plasticizing zone, the bonding takes place with a small anchoring of the harder material in the softer material. The presence of stainless-steel inclusions in aluminium is justified because the milling speeds were quite high (1250 rpm); other researchers who used lower speeds obtained no inclusions with similar processes (e.g., Sandnes, Lise (NTNU)).

The Interface between the two materials Is very well defined because of the fact that specimens extracted for tensile testing from outside the crater zone have validated the technology, which is also reflected in the microscopic analysis shown in Figure 10b [20,21,22,23].

## 4. FEM Simulation on AlMg3-X2CrNiMo17-12-2 Specimen

A numerical model was defined in Abaqus to assess the response of the entire tensile test specimen. As the tensile tests’ failure occurred in the base material, the considered analysis is useful for the assessment of joint elements and not necessarily the joining.

**Figure 11:**
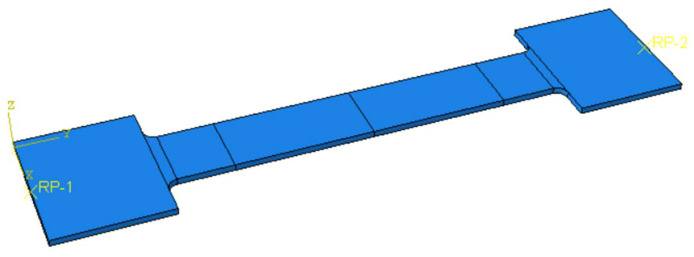
FEM part.

**Figure 12:**
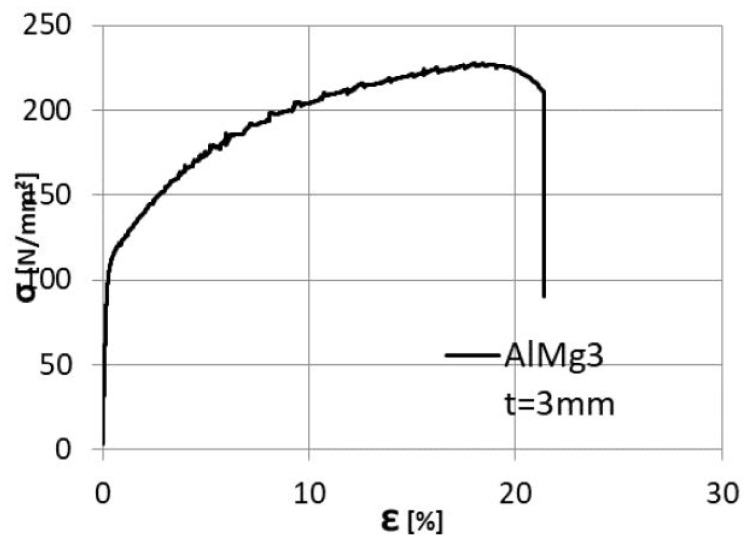
AlMg3 stress–strain curve.

The geometry of the tested specimen is presented in Figure 11, and it was modelled using volumetric elements as two separate identical parts. The two parts were assigned two different material constitutive laws. For the X2CrNiMo17-12-2, the nominal mechanical properties were defined as 200 Mpa, 500 Mpa, and 50% for the yield limit R_p0.2_; the tensile strength—R_m_ and elongation at fracture—A, respectively. The aluminium alloy, AlMg3, was defined using the properties determined from tests, considering 113 Mpa, 228 Mpa, and 24% for the yield stress R_p0.2_, tensile strength—R_m_, and elongation at fracture—A, as shown in Figure 12.

**Figure 13:**
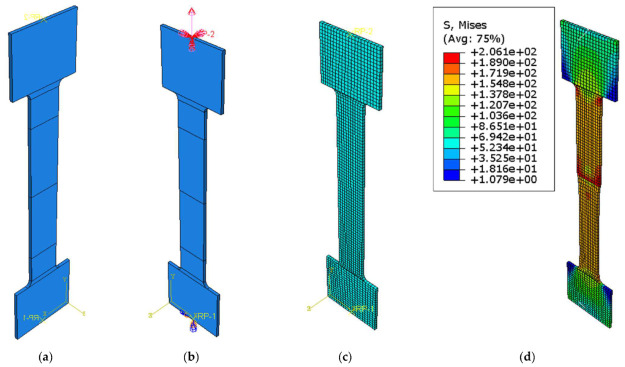
(**a**) Numerical model of the specimen, (**b**) boundary conditions, (**c**) finite element mesh, and (**d**) stress distribution in the specimen.

The two parts were assembled using the *TIE* command, resulting in the specimen presented in Figure 13a, where the division lines in the parallel length represent the monitored distance of the tensile test extensometer. Rigid body constraints were defined at the ends of the specimen using two reference points. The reference points were used to define the boundary conditions. A fixed support was defined at one end, while a displacement of 30 mm was defined for the other end; Figure 13b.

The C3D8R (8-node linear brick, reduced integration, and hourglass control) finite element type was chosen for the analysis. It had an approximate size of 3 mm.

**Figure 14:**
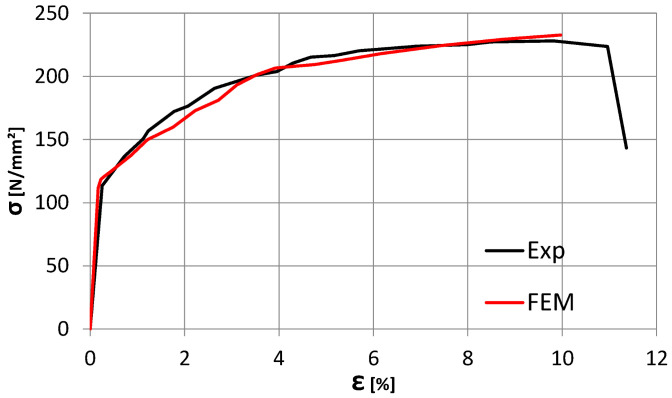
Comparison between the Experimental and Finite element analysis of the specimen. The two curves show a good similarity with a small discrepancy observed in the elastic range of the specimen behaviour.

With the presented data above, a static analysis step was defined, resulting in the response of the specimen presented in Figure 14.

## 5. Other Examples of Joints Obtained on Dissimilar Material on FSW Principle

There are references to various attempts to obtain dissimilar joints based on aluminium alloys using the friction stir welding process (FSW). For example, attempts have been made with good results to obtain a joint between the AlSi1MgMn alloy (EN AW 6082) and some pure copper plates using a conventional installation [12].

The pictures below present some tests performed on different materials and material combinations in the same welding lab and team.

**Figure 15:**
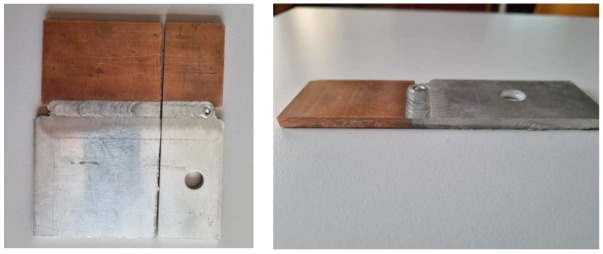
A joint of the AlMg3 + Cu alloy.

A dissimilar joint was obtained by joining an alloy of Cu with AlMg3 (Figure 15), with the following technology.

The joining parameters were the following:

- The rotation speed of the tool—1000 rpm.
- The advance speed of the tool—0.28 m/min.
- The length of the welding pin—2.7 mm

All of these examples are joints made with a modified milling machine on which a pin has been installed to obtain a process such as FSW in the welding lab in the Department of Materials and Manufacturing Engineering at the Faculty of Mechanics in Timisoara. All the experiments used the same length of welding pin (approximately 2.7 mm).

Microscopic structure of specimens:

In the case of the Al-Cu bonding in the microscopic images, the interaction between the two materials is better observed. From the images, the aluminium is the most engaged because the pin was on the aluminium side; moreover, in this joint, the aluminium only reached the plasticising phase. The darker-coloured aluminium zone is the zone where the aluminium has plasticised, i.e., the thermo-mechanically affected zone (TMAZ).

**Figure 16:**
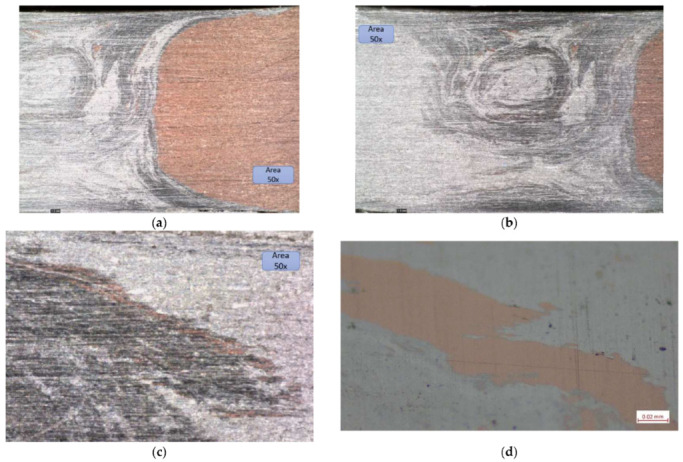
(**a**) the interface between the two alloys, (**b**) aluminium zone where there are few CU inclusions, (**c**) area at the base of the joint, and (**d**) microscopic image at 500×.

The nugget zone (NZ) can be observed in Figure 16b,c; in the last figure, from the base of the joint, some CU inclusions can be observed. In this case of the joint, the inclusions are more linear and follow the route of the pin.

In this joint, it is observed that there are no specific imperfections that could occur in these areas (insufficiently stirred root, voids, cracks, tunnel defects, surface lack of fill, seam underfill, blisters or surface galling, excessive surface flash formation, and irregular seam surface).

## 6. Conclusions

**Figure 17:**
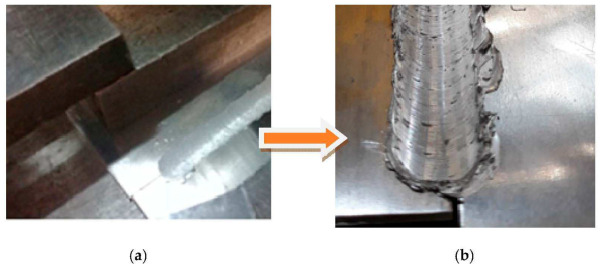
End areas of the joint. (**a**) heterogeneous joining obtaoned with FSW process. (**b**) flashing at the end of the joining.

This study, carried out on a specific type of joint, shows that the joint is sustainable; only two out of six specimens failed in tensile tests. The two specimens failed because they were sampled from the end areas of the obtained specimen, i.e., the areas where the pin enters and exits (Figure 17).

The optimised procedure joined the specimens on both sides; in this way, the area with the lack of penetration disappeared. Before this study, the joining technology was tested and validated on two different materials, and on AlMg3 sheets only after optimising the welding technology and the infrastructure used on previous studies.

In the microstructure of the weld, in the thermally influenced zone, were observed some dark points; however, the conclusion was that the particles of stainless-steel X2CrNiMo17-12-2 alloys were incorporated into the aluminium structure. The X2CrNiMo17-12-2 particles were distributed on opposite sides by the stirring forces of the tool.

FSW demonstrated that it can be a productive and, at the same time, relatively inexpensive welding process that does not require the use of filler material, while saving the price of a professional plant.

The much lower cost of the milling machine compared to a dedicated FSW machine, together with the possibility of using several types of pin geometries and obtaining high quality joints, are strong arguments in favour of the development of high performance-dedicated FSW-joining machines based on conventional milling machines. The research should continue to improve the flexibility of the milling machine by adding more motors, stiffening it, and developing force control solutions to prevent damage to the equipment. The research is also intended to be continued by modifying the length of the pin and the number of milling cutter revolutions, as both factors can generate a non-union zone in the joint.

This innovative solid-state method of joining opens a whole new range of welding possibilities—the low melting points of soft non-ferrous metals no longer pose a problem and, in the future, maybe such joints can be also used in civil engineering fields.

Even if FSW leads to low defect rates, the process must be controlled, and the joint must ensure that there are no defects that could compromise the integrity of the tool. One of the challenges of FSW is detecting defects in welded joints, as some of the defects associated with FSW are difficult to observe non-destructively.

The aesthetics of the weld are very satisfactory, and are without the need for further processing. Compared to other joining processes, the preparation of the parts is much easier, and in some cases, not even necessary.

Deformations resulting from the welding process are very small or almost non-existent.

As the number of specimens produced has been quite small, the research will continue in this direction because the joint can be obtained relatively quickly, without too many preparation operations and with the help of infrastructures that are quite low cost compared to machines designed exclusively for FSW.

Comparing them metallographically, both joints are obtained in the same laboratory, and it is observed that the microstructures provided are adequate and show a correct dissimilar joint without specific defects of the FSW process (wormhole, kiss bonding, etc).

The numerical model was defined to assess the response of the entire tensile test specimen. As the tensile test failure occurred with four specimens in the base material, the considered analysis is useful for the assessment of joint elements and not necessarily the joining.

## Figures and Tables

**Table 1:** Chemical composition of AlMg3 [11].

Charge	S37106411	Min.	
Si	0.25	0.4	Obs: 0.10–0.06 Mn + Cr
Fe	0.33	0.4	
Cu	0.059	0.1	
Mn	0.26	0.5	
Mg	2.8	2.6 0.3	
Cr	0.035	0.05	Each max.: 0.05
Ni	0.01	0.2	
Zn	0.04	0.15	
Ti	0.016	0.05	All max.: 0.15
Ga	0.011	0.05	
V	0.015		
Al	96.17	Difference	

**Table 2:** Mechanical properties of AlMg3 and X2CrNiMo17-12-2 [11].

Mechanical Properties of AlMg3	Value
R_m_ (MPa)—min. values from Standard (EN 485)	190
R_m_ (MPa)—max. values from Standard (EN 485)	240
R_m_ (MPa)—measured values	212
Rp_0.2_ (MPa)—min. values from Standard (EN 485)	80
Rp_0.2_ (MPa)—measured values	128
Elong. % 50 mm—values from Standard	16
Elong. % 50 mm—measured values	26.5
E[MPa]	200,000
Elong. % -mm	45
R_m_ (MPa)	500
Rp_0.2_ (MPa)	200
ρ (g/cm^3^)	8
C%	0.03
Si%	1.00
Mn%	2.00
P%	0.045
S%	0.015
Cr%	16.5
Mo%	2.5
Ni%	10–13
N%	0.11

**Table 3:** Comparison between the milling machine and the equipment used for FSW welding.

	Milling Machine	FSW Equipment	Parallel Robot	Articulated Robot
Flexibility	Low	Low/Medium	High	High
Cost	Medium	High	High	Low
Stiffness	High	High	High	Low
Work volume	Medium	Medium	Low	High
Setting time	Low	High	Medium	Medium
Number of programs	Low	Medium	High	High
Capacity of producing complex welds	Low	Medium	High	High
Type of control	Movement	Movement/force	Movement	Movement

**Table 4:** Chemical composition of the pin’s alloy % (56SiCr7, in accordance with SR EN 10089:2003 [11].

Chemical Composition	Value
C%	0.52–0.6
Si%	1.6–2.0
Mn%	0.7–1.0
P%	max. 0.25
S%	max. 0.25
Cr%	0.20–0.45
Cu + 10 Sn	<0.6

**Table 5:** Tensile test results.

Specimen Identification Code	Rp_0.2_	R_m_	A_t_
1 A-I	150.78	227.00	8.218
2 A-I	159.47	230.32	11.43
3 A-I	156.72	229.30	10.90
4 A-I	143.64	228.52	10.70
5 A-I	143.14	227.84	10.25
6 A-I	149.29	227.20	8.73

## Data Availability

The data presented in this study are available on request from the corresponding author.
